# (Acetyl­acetonato-κ^2^ *O*,*O*′)bis­[5-meth­oxy-2-(naphth[1,2-*d*][1,3]oxazol-2-yl)phenyl-κ^2^ *C* ^1^,*N*]iridium(III)

**DOI:** 10.1107/S1600536811035690

**Published:** 2011-09-14

**Authors:** Song Li, Guo-Jie Yin, Shi-Min Wang, Yuan-Yuan Zhou

**Affiliations:** a Institute of Electric Power, North China University of Water Resources and Electric Power, 450011, Zhengzhou, People’s Republic of China; b Department of Environment Engineering and Chemistry, Luoyang Institute of Science and Technology, 471023, Luoyang, People’s Republic of China; c Chemistry Department, Zhengzhou University, 450052, Zhengzhou, People’s Republic of China; d Institute of Environmental & Municipal Engineering, North China University of Water Resources and Electric Power, 450011, Zhengzhou, People’s Republic of China

## Abstract

In the title compound, [Ir(C_18_H_12_NO_2_)_2_(C_5_H_7_O_2_)], the Ir atom is *O*,*O*′-chelated by the acetyl­acetonate group and *C*,*N*-chelated by the 2-aryl­naphth[1,2-*d*]oxazole groups. The six-coordinate metal atom displays a distorted octa­hedral geometry. Intra­molecular C—H⋯O hydrogen bonds occur. In the crystal, inter­molecular C—H⋯O hydrogen bonds link the mol­ecules into columns parallel to the *b* axis.

## Related literature

For the syntheses and reactions of some 2-aryl­naphth[1,2-*d*]oxazole derivatives, see: Abbady (1979 ▶). For the syntheses and characterization of phospho­rescent cyclo­metalated iridium complexes, see: Lamansky *et al.* (2001 ▶).
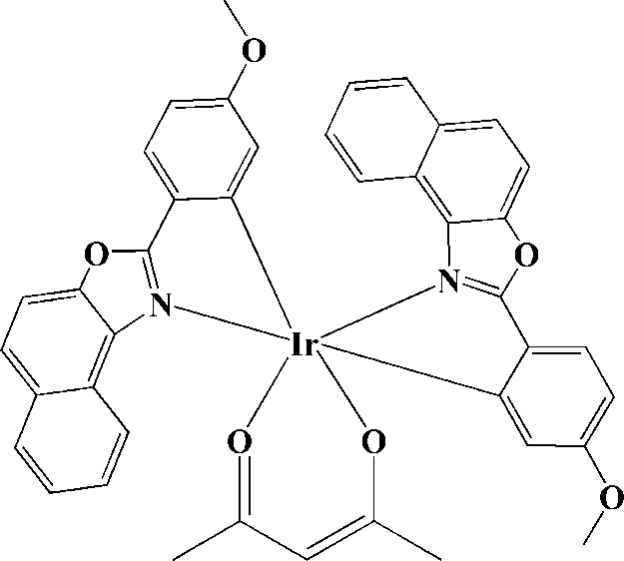


## Experimental

### 

#### Crystal data

[Ir(C_18_H_12_NO_2_)_2_(C_5_H_7_O_2_)]
*M* *_r_* = 839.88
Monoclinic, 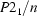

*a* = 16.618 (3) Å
*b* = 11.455 (2) Å
*c* = 18.993 (4) Å
β = 114.01 (3)°
*V* = 3302.5 (13) Å^3^
*Z* = 4
Mo *K*α radiation
μ = 4.10 mm^−1^
*T* = 293 K
0.30 × 0.20 × 0.20 mm

#### Data collection

Bruker SMART CCD area detector diffractometer
Absorption correction: multi-scan (*SADABS*; Sheldrick, 2004 ▶) *T*_min_ = 0.373, *T*_max_ = 0.495
40039 measured reflections
7866 independent reflections
7275 reflections with *I* > 2σ(*I*)
*R*_int_ = 0.042

#### Refinement

*R*[*F* ^2^ > 2σ(*F* ^2^)] = 0.039
*wR*(*F* ^2^) = 0.093
*S* = 1.04
7866 reflections
452 parameters
H-atom parameters not refined
Δρ_max_ = 1.19 e Å^−3^
Δρ_min_ = −0.79 e Å^−3^

### 

Data collection: *SMART* (Bruker, 2001 ▶); cell refinement: *SAINT* (Bruker, 2001 ▶); data reduction: *SAINT*; program(s) used to solve structure: *SHELXS97* (Sheldrick, 2008 ▶); program(s) used to refine structure: *SHELXL97* (Sheldrick, 2008 ▶); molecular graphics: *SHELXTL* (Sheldrick, 2008 ▶); software used to prepare material for publication: *SHELXTL*.

## Supplementary Material

Crystal structure: contains datablock(s) global, I. DOI: 10.1107/S1600536811035690/rz2632sup1.cif

Structure factors: contains datablock(s) I. DOI: 10.1107/S1600536811035690/rz2632Isup2.hkl

Additional supplementary materials:  crystallographic information; 3D view; checkCIF report

## Figures and Tables

**Table 1:** Selected bond lengths (Å)

Ir1—C1	1.997 (4)
Ir1—C19	2.004 (4)
Ir1—N1	2.088 (3)
Ir1—N2	2.109 (3)
Ir1—O6	2.144 (3)
Ir1—O5	2.156 (3)

**Table 2:** Hydrogen-bond geometry (Å, °)

*D*—H⋯*A*	*D*—H	H⋯*A*	*D*⋯*A*	*D*—H⋯*A*
C15—H15*A*⋯O6	0.93	2.24	3.077 (5)	150
C33—H33*A*⋯O5	0.93	2.22	3.137 (6)	170
C23—H23*A*⋯O6^i^	0.93	2.54	3.419 (6)	157
C27—H27*A*⋯O2^ii^	0.93	2.55	3.206 (6)	128

Symmetry codes: (i) 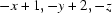; (ii) 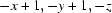.

**Fig. 1.**
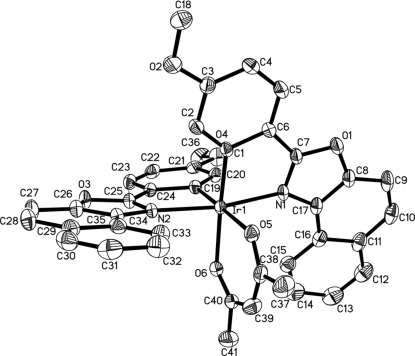
The molecular structure of the title complex with displacement ellipsoids drawn at the 30% probability level. Hydrogen atoms are omitted for clarity.

## supplementary crystallographic information

### Comment

According to the study of Lamansky's group in 2001 (Lamansky *et al.*, 2001), the luminous wavelength of complexes would change as the the conjugated system of (C—N) changed. Therefore, the arylnaphthoxazoles ligand was choosed to regulate luminous wavelength of phosphorescent materials, leading to get better electrophosphorescent materials.

The title complex is a mononuclear iridium(III) complex (Fig. 1), in which the environment around the Ir^III^ ion is a distorted octahedral coordination geometry, the coordination conformation of the C, N and O atoms of the ligands adopt the *cis*-, *trans*- and *cis*- respectively, which is consistent with the similar reported complexes (Lamansky *et al.*, 2001). It can be illustrated from the figure that the carbon-metal bond is formed between Ir^III^ ion and the carbon atom on the benzene ring rather than the C atom on the naphthalene ring. It shows from Table 1 that the increase of the bond distance from Ir—C to Ir—N and Ir—O is caused by the increase of the covalent component between the coordination atoms from C to N and O of which the electronegativity decreases gradually. Moreover, there are two five-membered rings formed (Ir1/C1/C6/C7/N1 and Ir1/C19/C24/C25/N2), the average deviation of which are 0.0186 Å and 0.0387 Å, and the dihedral angle they form with their adjacent benzene rings (C1–C6) and (C19–C24) are 3.5 (2)° and 4.9 (3)° respectively. The dihedral angle with their adjacent oxazole heterocycle (N1/O1/C7–17) and (N2/O3/C25–35) are 9.0 (2)° and 8.0 (2)° respectively. The molecular comformation is stabilized by intramolecular C—H···O hydrogen bonds (Table 2). In the crystal structure, intermolecular C—H···O hydrogen bonds (Table 2) link molecules into columns parallel to the *b* axis.

### Experimental

The ligand 2-arylnaphth[1,2-*d*]oxazole was prepared according to the literature (Abbady, 1979). The ligand (0.61 g, 2.2 mmol) and IrCl_3_.3H_2_O (0.35 g, 1 mmol) were added to 20 ml 2-ethoxyethanol:H_2_O (3:1, *v*/*v*) solution under inert gas atmosphere at 393 K for 24 h, and then the intermediate product, acetylacetonate (10 ml) and Na_2_CO_3_ (1.06 g, 10 mmol) were refluxed for 12 h. After cooling to room temperature, the coloured precipitate was filtered and washed with ethanol and water. The crude product was flash chromatographed using a silica/dichloromethane column to yield *ca*. 40% of the pure title compound after solvent evaporation and drying.

### Refinement

H atoms were positioned geometrically and refined using a riding model with C—H =0.93–0.96 Å and with *U*_iso_(H) = 1.2 (1.5 for methyl groups) times *U*_eq_(C).

### Crystal data

**Table:**

[Ir(C_18_H_12_NO_2_)_2_(C_5_H_7_O_2_)]	*F*(000) = 1664
*M**_r_* = 839.88	*D*_x_ = 1.689 Mg m^−^^3^
Monoclinic, *P*2_1_/*n*	Mo *K*α radiation, λ = 0.71073 Å
Hall symbol: -P 2yn	Cell parameters from 168 reflections
*a* = 16.618 (3) Å	θ = 2.5–26.0°
*b* = 11.455 (2) Å	µ = 4.10 mm^−^^1^
*c* = 18.993 (4) Å	*T* = 293 K
β = 114.01 (3)°	Prismatic, yellow
*V* = 3302.5 (13) Å^3^	0.30 × 0.20 × 0.20 mm
*Z* = 4	

### Data collection

**Table:**

Bruker SMART CCD area detector diffractometer	7866 independent reflections
Radiation source: fine-focus sealed tube	7275 reflections with *I* > 2σ(*I*)
graphite	*R*_int_ = 0.042
phi and ω scans	θ_max_ = 27.9°, θ_min_ = 2.2°
Absorption correction: multi-scan (*SADABS*; Sheldrick, 2004)	*h* = −21→21
*T*_min_ = 0.373, *T*_max_ = 0.495	*k* = −15→14
40039 measured reflections	*l* = −24→24

### Refinement

**Table:**

Refinement on *F*^2^	Secondary atom site location: difference Fourier map
Least-squares matrix: full	Hydrogen site location: inferred from neighbouring sites
*R*[*F*^2^ > 2σ(*F*^2^)] = 0.039	H-atom parameters not refined
*wR*(*F*^2^) = 0.093	*w* = 1/[σ^2^(*F*_o_^2^) + (0.049*P*)^2^ + 2.9*P*] where *P* = (*F*_o_^2^ + 2*F*_c_^2^)/3
*S* = 1.04	(Δ/σ)_max_ = 0.001
7866 reflections	Δρ_max_ = 1.19 e Å^−^^3^
452 parameters	Δρ_min_ = −0.79 e Å^−^^3^
0 restraints	Extinction correction: *SHELXL*, Fc^*^=kFc[1+0.001xFc^2^λ^3^/sin(2θ)]^-1/4^
Primary atom site location: structure-invariant direct methods	Extinction coefficient: 0.000124

### Special details

**Table:**

Geometry. All e.s.d.'s (except the e.s.d. in the dihedral angle between two l.s. planes) are estimated using the full covariance matrix. The cell e.s.d.'s are taken into account individually in the estimation of e.s.d.'s in distances, angles and torsion angles; correlations between e.s.d.'s in cell parameters are only used when they are defined by crystal symmetry. An approximate (isotropic) treatment of cell e.s.d.'s is used for estimating e.s.d.'s involving l.s. planes.
Refinement. Refinement of *F*^2^ against ALL reflections. The weighted *R*-factor *wR* and goodness of fit *S* are based on *F*^2^, conventional *R*-factors *R* are based on *F*, with *F* set to zero for negative *F*^2^. The threshold expression of *F*^2^ > σ(*F*^2^) is used only for calculating *R*-factors(gt) *etc*. and is not relevant to the choice of reflections for refinement. *R*-factors based on *F*^2^ are statistically about twice as large as those based on *F*, and *R*- factors based on ALL data will be even larger.

### Fractional atomic coordinates and isotropic or equivalent isotropic displacement parameters (Å^2^)

**Table:**

	*x*	*y*	*z*	*U*_iso_*/*U*_eq_	
Ir1	0.283405 (9)	0.883603 (13)	0.016855 (9)	0.03107 (8)	
C1	0.2104 (2)	0.7556 (4)	−0.0502 (2)	0.0333 (9)	
C2	0.2356 (3)	0.6436 (4)	−0.0595 (3)	0.0400 (10)	
H2A	0.2948	0.6231	−0.0358	0.048*	
C3	0.1744 (3)	0.5607 (4)	−0.1037 (3)	0.0433 (10)	
C4	0.0850 (3)	0.5876 (5)	−0.1400 (3)	0.0497 (12)	
H4A	0.0447	0.5323	−0.1701	0.060*	
C5	0.0575 (3)	0.6967 (4)	−0.1305 (3)	0.0485 (12)	
H5A	−0.0020	0.7160	−0.1540	0.058*	
C6	0.1186 (3)	0.7791 (4)	−0.0857 (3)	0.0390 (10)	
C7	0.0990 (3)	0.8925 (4)	−0.0681 (3)	0.0383 (10)	
C8	0.0285 (3)	1.0503 (4)	−0.0648 (3)	0.0442 (11)	
C9	−0.0388 (4)	1.1294 (5)	−0.0754 (3)	0.0572 (15)	
H9A	−0.0977	1.1110	−0.1038	0.069*	
C10	−0.0131 (4)	1.2354 (5)	−0.0418 (3)	0.0608 (15)	
H10A	−0.0560	1.2899	−0.0451	0.073*	
C11	0.0772 (4)	1.2659 (5)	−0.0016 (3)	0.0545 (13)	
C12	0.1028 (5)	1.3789 (5)	0.0288 (4)	0.0690 (18)	
H12A	0.0595	1.4319	0.0269	0.083*	
C13	0.1882 (5)	1.4127 (5)	0.0608 (4)	0.0721 (18)	
H13A	0.2030	1.4877	0.0806	0.086*	
C14	0.2539 (4)	1.3346 (5)	0.0639 (3)	0.0636 (15)	
H14A	0.3123	1.3592	0.0832	0.076*	
C15	0.2333 (3)	1.2218 (4)	0.0388 (3)	0.0488 (11)	
H15A	0.2782	1.1701	0.0430	0.059*	
C16	0.1460 (3)	1.1836 (4)	0.0070 (3)	0.0457 (11)	
C17	0.1165 (3)	1.0700 (4)	−0.0234 (3)	0.0403 (10)	
C18	0.1518 (4)	0.3653 (4)	−0.1511 (4)	0.0623 (15)	
H18A	0.1853	0.2965	−0.1497	0.093*	
H18B	0.1201	0.3897	−0.2035	0.093*	
H18C	0.1108	0.3484	−0.1285	0.093*	
C19	0.3098 (2)	0.9591 (3)	−0.0666 (2)	0.0326 (8)	
C20	0.2626 (3)	1.0416 (4)	−0.1207 (2)	0.0371 (9)	
H20A	0.2070	1.0631	−0.1245	0.045*	
C21	0.2958 (3)	1.0938 (4)	−0.1699 (3)	0.0395 (10)	
C22	0.3789 (3)	1.0652 (4)	−0.1654 (3)	0.0437 (10)	
H22A	0.4014	1.1023	−0.1970	0.052*	
C23	0.4275 (3)	0.9816 (4)	−0.1140 (3)	0.0423 (10)	
H23A	0.4825	0.9599	−0.1115	0.051*	
C24	0.3936 (3)	0.9294 (4)	−0.0655 (2)	0.0353 (9)	
C25	0.4352 (3)	0.8390 (4)	−0.0116 (3)	0.0375 (9)	
C26	0.5213 (3)	0.6961 (4)	0.0445 (3)	0.0434 (10)	
C27	0.5894 (3)	0.6147 (5)	0.0656 (4)	0.0567 (14)	
H27A	0.6318	0.6163	0.0453	0.068*	
C28	0.5899 (3)	0.5331 (5)	0.1174 (3)	0.0580 (14)	
H28A	0.6337	0.4763	0.1327	0.070*	
C29	0.5257 (3)	0.5314 (4)	0.1492 (3)	0.0490 (12)	
C30	0.5291 (4)	0.4462 (5)	0.2044 (3)	0.0586 (14)	
H30A	0.5739	0.3908	0.2201	0.070*	
C31	0.4684 (4)	0.4443 (5)	0.2344 (3)	0.0605 (14)	
H31A	0.4715	0.3872	0.2702	0.073*	
C32	0.4011 (4)	0.5264 (5)	0.2124 (3)	0.0610 (14)	
H32A	0.3603	0.5246	0.2345	0.073*	
C33	0.3941 (4)	0.6101 (4)	0.1586 (3)	0.0484 (12)	
H33A	0.3479	0.6634	0.1435	0.058*	
C34	0.4560 (3)	0.6157 (4)	0.1264 (3)	0.0406 (10)	
C35	0.4572 (3)	0.7002 (4)	0.0720 (3)	0.0382 (9)	
C36	0.2760 (4)	1.2488 (5)	−0.2607 (3)	0.0625 (15)	
H36A	0.2304	1.3002	−0.2933	0.094*	
H36B	0.2972	1.2026	−0.2918	0.094*	
H36C	0.3236	1.2941	−0.2248	0.094*	
C37	0.2550 (4)	0.7829 (6)	0.2320 (3)	0.0692 (16)	
H37A	0.2206	0.7168	0.2053	0.104*	
H37B	0.2206	0.8310	0.2507	0.104*	
H37C	0.3067	0.7564	0.2747	0.104*	
C38	0.2818 (3)	0.8530 (5)	0.1775 (3)	0.0485 (12)	
C39	0.3321 (4)	0.9528 (5)	0.2054 (3)	0.0566 (13)	
H39A	0.3440	0.9733	0.2561	0.068*	
C40	0.3666 (3)	1.0257 (4)	0.1659 (3)	0.0436 (11)	
C41	0.4168 (4)	1.1320 (5)	0.2078 (4)	0.0654 (16)	
H41A	0.4372	1.1748	0.1748	0.098*	
H41B	0.4663	1.1081	0.2534	0.098*	
H41C	0.3788	1.1809	0.2220	0.098*	
O1	0.01686 (18)	0.9383 (3)	−0.09431 (19)	0.0458 (8)	
O2	0.2092 (2)	0.4554 (3)	−0.1090 (2)	0.0568 (9)	
O3	0.50843 (19)	0.7840 (3)	−0.0083 (2)	0.0459 (8)	
O4	0.2417 (2)	1.1741 (3)	−0.2197 (2)	0.0554 (9)	
O5	0.25520 (19)	0.8129 (3)	0.10959 (18)	0.0422 (7)	
O6	0.35994 (18)	1.0133 (3)	0.09775 (18)	0.0402 (7)	
N1	0.1604 (2)	0.9649 (3)	−0.0240 (2)	0.0357 (8)	
N2	0.4017 (2)	0.7958 (3)	0.0350 (2)	0.0332 (7)	

### Atomic displacement parameters (Å^2^)

**Table:**

	*U*^11^	*U*^22^	*U*^33^	*U*^12^	*U*^13^	*U*^23^
Ir1	0.02440 (10)	0.03730 (12)	0.03357 (11)	0.00402 (6)	0.01390 (7)	0.00183 (6)
C1	0.0253 (19)	0.045 (2)	0.031 (2)	0.0023 (17)	0.0132 (16)	0.0050 (17)
C2	0.030 (2)	0.049 (3)	0.038 (2)	0.0034 (19)	0.0104 (18)	0.0009 (19)
C3	0.040 (2)	0.043 (3)	0.051 (3)	0.000 (2)	0.024 (2)	−0.004 (2)
C4	0.036 (2)	0.052 (3)	0.055 (3)	−0.005 (2)	0.013 (2)	−0.010 (2)
C5	0.028 (2)	0.057 (3)	0.055 (3)	0.005 (2)	0.011 (2)	0.000 (2)
C6	0.029 (2)	0.046 (2)	0.041 (2)	0.0008 (18)	0.0132 (18)	−0.0030 (19)
C7	0.026 (2)	0.050 (3)	0.040 (2)	0.0080 (17)	0.0146 (18)	0.0052 (19)
C8	0.035 (2)	0.056 (3)	0.047 (3)	0.014 (2)	0.023 (2)	0.012 (2)
C9	0.041 (3)	0.076 (4)	0.062 (3)	0.029 (3)	0.029 (3)	0.026 (3)
C10	0.062 (3)	0.062 (4)	0.074 (4)	0.032 (3)	0.043 (3)	0.020 (3)
C11	0.060 (3)	0.054 (3)	0.059 (3)	0.025 (3)	0.033 (3)	0.015 (2)
C12	0.087 (5)	0.050 (3)	0.075 (4)	0.031 (3)	0.038 (4)	0.009 (3)
C13	0.097 (5)	0.049 (3)	0.073 (4)	0.011 (3)	0.036 (4)	0.003 (3)
C14	0.075 (4)	0.046 (3)	0.064 (4)	0.009 (3)	0.023 (3)	0.004 (3)
C15	0.053 (3)	0.047 (3)	0.048 (3)	0.012 (2)	0.022 (2)	0.006 (2)
C16	0.052 (3)	0.049 (3)	0.043 (3)	0.015 (2)	0.026 (2)	0.011 (2)
C17	0.035 (2)	0.047 (3)	0.045 (3)	0.015 (2)	0.023 (2)	0.012 (2)
C18	0.065 (4)	0.045 (3)	0.076 (4)	−0.002 (3)	0.028 (3)	−0.009 (3)
C19	0.0285 (19)	0.035 (2)	0.037 (2)	−0.0011 (16)	0.0169 (17)	0.0007 (17)
C20	0.037 (2)	0.040 (2)	0.039 (2)	0.0033 (18)	0.0199 (18)	0.0045 (18)
C21	0.048 (3)	0.039 (2)	0.033 (2)	0.0000 (19)	0.019 (2)	−0.0004 (18)
C22	0.051 (3)	0.050 (3)	0.042 (3)	−0.006 (2)	0.031 (2)	−0.002 (2)
C23	0.037 (2)	0.051 (3)	0.047 (3)	0.001 (2)	0.025 (2)	−0.001 (2)
C24	0.030 (2)	0.042 (2)	0.038 (2)	0.0003 (18)	0.0185 (18)	−0.0013 (18)
C25	0.0275 (19)	0.043 (2)	0.043 (2)	0.0061 (18)	0.0157 (18)	−0.0017 (19)
C26	0.033 (2)	0.047 (3)	0.051 (3)	0.0080 (19)	0.018 (2)	0.001 (2)
C27	0.039 (3)	0.069 (4)	0.066 (4)	0.020 (2)	0.025 (3)	0.007 (3)
C28	0.042 (3)	0.060 (3)	0.066 (4)	0.022 (2)	0.017 (3)	0.007 (3)
C29	0.046 (3)	0.046 (3)	0.046 (3)	0.012 (2)	0.010 (2)	0.001 (2)
C30	0.056 (3)	0.047 (3)	0.055 (3)	0.015 (2)	0.005 (3)	0.007 (2)
C31	0.076 (4)	0.046 (3)	0.051 (3)	0.004 (3)	0.018 (3)	0.006 (2)
C32	0.066 (3)	0.060 (3)	0.061 (4)	−0.002 (3)	0.030 (3)	0.007 (3)
C33	0.049 (3)	0.047 (3)	0.052 (3)	0.003 (2)	0.023 (2)	0.002 (2)
C34	0.039 (2)	0.038 (2)	0.039 (2)	0.0028 (18)	0.010 (2)	0.0010 (18)
C35	0.027 (2)	0.044 (2)	0.040 (2)	0.0061 (18)	0.0105 (17)	−0.0017 (19)
C36	0.089 (4)	0.058 (3)	0.049 (3)	0.006 (3)	0.037 (3)	0.015 (3)
C37	0.076 (4)	0.091 (4)	0.052 (3)	0.007 (3)	0.039 (3)	0.014 (3)
C38	0.042 (3)	0.066 (3)	0.044 (3)	0.020 (2)	0.024 (2)	0.009 (2)
C39	0.061 (3)	0.066 (3)	0.044 (3)	0.005 (3)	0.022 (2)	−0.006 (2)
C40	0.034 (2)	0.050 (3)	0.042 (3)	0.010 (2)	0.0107 (19)	−0.008 (2)
C41	0.060 (4)	0.063 (4)	0.065 (4)	−0.001 (3)	0.017 (3)	−0.016 (3)
O1	0.0278 (15)	0.058 (2)	0.0507 (19)	0.0074 (14)	0.0150 (14)	0.0050 (16)
O2	0.0469 (19)	0.0436 (19)	0.074 (3)	0.0006 (15)	0.0181 (18)	−0.0136 (17)
O3	0.0318 (15)	0.057 (2)	0.056 (2)	0.0130 (14)	0.0247 (14)	0.0084 (16)
O4	0.059 (2)	0.055 (2)	0.057 (2)	0.0100 (17)	0.0291 (18)	0.0214 (17)
O5	0.0372 (16)	0.0557 (19)	0.0395 (17)	0.0050 (14)	0.0215 (14)	0.0086 (14)
O6	0.0296 (14)	0.0452 (17)	0.0457 (18)	0.0019 (13)	0.0151 (13)	−0.0041 (14)
N1	0.0253 (16)	0.044 (2)	0.042 (2)	0.0092 (15)	0.0175 (15)	0.0079 (16)
N2	0.0238 (16)	0.0381 (19)	0.0375 (19)	0.0057 (14)	0.0125 (14)	0.0002 (15)

### Geometric parameters (Å, °)

**Table:**

Ir1—C1	1.997 (4)	C21—O4	1.364 (5)
Ir1—C19	2.004 (4)	C21—C22	1.387 (6)
Ir1—N1	2.088 (3)	C22—C23	1.371 (6)
Ir1—N2	2.109 (3)	C22—H22A	0.9300
Ir1—O6	2.144 (3)	C23—C24	1.393 (6)
Ir1—O5	2.156 (3)	C23—H23A	0.9300
C1—C2	1.383 (6)	C24—C25	1.423 (6)
C1—C6	1.420 (5)	C25—N2	1.318 (5)
C2—C3	1.395 (6)	C25—O3	1.348 (5)
C2—H2A	0.9300	C26—C35	1.365 (6)
C3—O2	1.359 (5)	C26—O3	1.374 (6)
C3—C4	1.394 (6)	C26—C27	1.393 (6)
C4—C5	1.367 (7)	C27—C28	1.355 (8)
C4—H4A	0.9300	C27—H27A	0.9300
C5—C6	1.394 (6)	C28—C29	1.426 (7)
C5—H5A	0.9300	C28—H28A	0.9300
C6—C7	1.413 (6)	C29—C30	1.416 (7)
C7—N1	1.317 (6)	C29—C34	1.433 (6)
C7—O1	1.354 (5)	C30—C31	1.345 (8)
C8—C17	1.369 (6)	C30—H30A	0.9300
C8—O1	1.382 (6)	C31—C32	1.389 (8)
C8—C9	1.388 (6)	C31—H31A	0.9300
C9—C10	1.356 (8)	C32—C33	1.371 (7)
C9—H9A	0.9300	C32—H32A	0.9300
C10—C11	1.423 (8)	C33—C34	1.397 (7)
C10—H10A	0.9300	C33—H33A	0.9300
C11—C12	1.410 (8)	C34—C35	1.421 (6)
C11—C16	1.440 (6)	C35—N2	1.420 (5)
C12—C13	1.354 (10)	C36—O4	1.423 (6)
C12—H12A	0.9300	C36—H36A	0.9600
C13—C14	1.394 (9)	C36—H36B	0.9600
C13—H13A	0.9300	C36—H36C	0.9600
C14—C15	1.371 (7)	C37—C38	1.514 (7)
C14—H14A	0.9300	C37—H37A	0.9600
C15—C16	1.396 (7)	C37—H37B	0.9600
C15—H15A	0.9300	C37—H37C	0.9600
C16—C17	1.427 (7)	C38—O5	1.267 (6)
C17—N1	1.410 (5)	C38—C39	1.388 (8)
C18—O2	1.412 (6)	C39—C40	1.393 (7)
C18—H18A	0.9600	C39—H39A	0.9300
C18—H18B	0.9600	C40—O6	1.261 (5)
C18—H18C	0.9600	C40—C41	1.507 (7)
C19—C20	1.381 (6)	C41—H41A	0.9600
C19—C24	1.426 (5)	C41—H41B	0.9600
C20—C21	1.399 (6)	C41—H41C	0.9600
C20—H20A	0.9300		
			
C1—Ir1—C19	94.82 (16)	C23—C22—C21	119.4 (4)
C1—Ir1—N1	80.88 (15)	C23—C22—H22A	120.3
C19—Ir1—N1	90.57 (14)	C21—C22—H22A	120.3
C1—Ir1—N2	92.18 (14)	C22—C23—C24	119.4 (4)
C19—Ir1—N2	81.11 (15)	C22—C23—H23A	120.3
N1—Ir1—N2	168.72 (13)	C24—C23—H23A	120.3
C1—Ir1—O6	174.57 (14)	C23—C24—C25	125.4 (4)
C19—Ir1—O6	90.60 (14)	C23—C24—C19	122.9 (4)
N1—Ir1—O6	99.37 (13)	C25—C24—C19	111.6 (4)
N2—Ir1—O6	88.39 (12)	N2—C25—O3	114.4 (4)
C1—Ir1—O5	88.16 (14)	N2—C25—C24	122.9 (4)
C19—Ir1—O5	176.47 (14)	O3—C25—C24	122.6 (4)
N1—Ir1—O5	88.03 (12)	C35—C26—O3	109.8 (4)
N2—Ir1—O5	100.70 (12)	C35—C26—C27	125.6 (5)
O6—Ir1—O5	86.43 (12)	O3—C26—C27	124.6 (4)
C2—C1—C6	116.0 (4)	C28—C27—C26	115.8 (5)
C2—C1—Ir1	128.7 (3)	C28—C27—H27A	122.1
C6—C1—Ir1	114.8 (3)	C26—C27—H27A	122.1
C1—C2—C3	121.5 (4)	C27—C28—C29	122.2 (5)
C1—C2—H2A	119.2	C27—C28—H28A	118.9
C3—C2—H2A	119.2	C29—C28—H28A	118.9
O2—C3—C4	124.0 (4)	C30—C29—C28	120.8 (5)
O2—C3—C2	114.9 (4)	C30—C29—C34	118.2 (5)
C4—C3—C2	121.1 (4)	C28—C29—C34	121.0 (5)
C5—C4—C3	118.9 (5)	C31—C30—C29	120.9 (5)
C5—C4—H4A	120.5	C31—C30—H30A	119.6
C3—C4—H4A	120.5	C29—C30—H30A	119.6
C4—C5—C6	119.9 (4)	C30—C31—C32	120.9 (5)
C4—C5—H5A	120.0	C30—C31—H31A	119.6
C6—C5—H5A	120.0	C32—C31—H31A	119.6
C5—C6—C7	125.9 (4)	C33—C32—C31	120.7 (6)
C5—C6—C1	122.5 (4)	C33—C32—H32A	119.6
C7—C6—C1	111.7 (4)	C31—C32—H32A	119.6
N1—C7—O1	113.2 (4)	C32—C33—C34	120.3 (5)
N1—C7—C6	122.4 (4)	C32—C33—H33A	119.8
O1—C7—C6	124.4 (4)	C34—C33—H33A	119.8
C17—C8—O1	109.0 (4)	C33—C34—C35	125.5 (4)
C17—C8—C9	125.8 (5)	C33—C34—C29	119.0 (4)
O1—C8—C9	125.2 (5)	C35—C34—C29	115.5 (4)
C10—C9—C8	115.8 (5)	C26—C35—N2	106.7 (4)
C10—C9—H9A	122.1	C26—C35—C34	119.9 (4)
C8—C9—H9A	122.1	N2—C35—C34	133.4 (4)
C9—C10—C11	122.2 (5)	O4—C36—H36A	109.5
C9—C10—H10A	118.9	O4—C36—H36B	109.5
C11—C10—H10A	118.9	H36A—C36—H36B	109.5
C12—C11—C10	121.4 (5)	O4—C36—H36C	109.5
C12—C11—C16	117.5 (5)	H36A—C36—H36C	109.5
C10—C11—C16	121.1 (5)	H36B—C36—H36C	109.5
C13—C12—C11	122.2 (5)	C38—C37—H37A	109.5
C13—C12—H12A	118.9	C38—C37—H37B	109.5
C11—C12—H12A	118.9	H37A—C37—H37B	109.5
C12—C13—C14	119.7 (6)	C38—C37—H37C	109.5
C12—C13—H13A	120.1	H37A—C37—H37C	109.5
C14—C13—H13A	120.1	H37B—C37—H37C	109.5
C15—C14—C13	120.6 (6)	O5—C38—C39	126.4 (5)
C15—C14—H14A	119.7	O5—C38—C37	115.2 (5)
C13—C14—H14A	119.7	C39—C38—C37	118.4 (5)
C14—C15—C16	121.0 (5)	C38—C39—C40	127.2 (5)
C14—C15—H15A	119.5	C38—C39—H39A	116.4
C16—C15—H15A	119.5	C40—C39—H39A	116.4
C15—C16—C17	126.0 (4)	O6—C40—C39	127.0 (5)
C15—C16—C11	118.8 (5)	O6—C40—C41	115.6 (5)
C17—C16—C11	115.1 (4)	C39—C40—C41	117.4 (5)
C8—C17—N1	106.9 (4)	C40—C41—H41A	109.5
C8—C17—C16	119.6 (4)	C40—C41—H41B	109.5
N1—C17—C16	133.5 (4)	H41A—C41—H41B	109.5
O2—C18—H18A	109.5	C40—C41—H41C	109.5
O2—C18—H18B	109.5	H41A—C41—H41C	109.5
H18A—C18—H18B	109.5	H41B—C41—H41C	109.5
O2—C18—H18C	109.5	C7—O1—C8	104.9 (3)
H18A—C18—H18C	109.5	C3—O2—C18	118.7 (4)
H18B—C18—H18C	109.5	C25—O3—C26	104.3 (3)
C20—C19—C24	115.4 (4)	C21—O4—C36	119.1 (4)
C20—C19—Ir1	130.0 (3)	C38—O5—Ir1	126.4 (3)
C24—C19—Ir1	114.4 (3)	C40—O6—Ir1	126.3 (3)
C19—C20—C21	122.0 (4)	C7—N1—C17	105.9 (3)
C19—C20—H20A	119.0	C7—N1—Ir1	110.1 (3)
C21—C20—H20A	119.0	C17—N1—Ir1	144.0 (3)
O4—C21—C22	124.2 (4)	C25—N2—C35	104.8 (3)
O4—C21—C20	115.1 (4)	C25—N2—Ir1	109.4 (3)
C22—C21—C20	120.7 (4)	C35—N2—Ir1	145.4 (3)
			
C19—Ir1—C1—C2	95.3 (4)	C29—C30—C31—C32	−0.5 (9)
N1—Ir1—C1—C2	−174.9 (4)	C30—C31—C32—C33	1.2 (9)
N2—Ir1—C1—C2	14.0 (4)	C31—C32—C33—C34	−1.5 (8)
O5—Ir1—C1—C2	−86.6 (4)	C32—C33—C34—C35	−178.1 (5)
C19—Ir1—C1—C6	−93.0 (3)	C32—C33—C34—C29	1.2 (7)
N1—Ir1—C1—C6	−3.2 (3)	C30—C29—C34—C33	−0.5 (7)
N2—Ir1—C1—C6	−174.2 (3)	C28—C29—C34—C33	179.5 (5)
O5—Ir1—C1—C6	85.1 (3)	C30—C29—C34—C35	178.8 (4)
C6—C1—C2—C3	2.4 (6)	C28—C29—C34—C35	−1.1 (7)
Ir1—C1—C2—C3	174.1 (3)	O3—C26—C35—N2	−0.4 (5)
C1—C2—C3—O2	178.6 (4)	C27—C26—C35—N2	−179.7 (5)
C1—C2—C3—C4	−0.4 (7)	O3—C26—C35—C34	178.6 (4)
O2—C3—C4—C5	−180.0 (5)	C27—C26—C35—C34	−0.7 (8)
C2—C3—C4—C5	−1.2 (8)	C33—C34—C35—C26	−179.8 (5)
C3—C4—C5—C6	0.4 (8)	C29—C34—C35—C26	0.9 (6)
C4—C5—C6—C7	−176.7 (5)	C33—C34—C35—N2	−1.1 (8)
C4—C5—C6—C1	1.8 (8)	C29—C34—C35—N2	179.6 (4)
C2—C1—C6—C5	−3.2 (7)	O5—C38—C39—C40	−3.1 (9)
Ir1—C1—C6—C5	−176.0 (4)	C37—C38—C39—C40	176.8 (5)
C2—C1—C6—C7	175.6 (4)	C38—C39—C40—O6	−1.4 (8)
Ir1—C1—C6—C7	2.7 (5)	C38—C39—C40—C41	177.9 (5)
C5—C6—C7—N1	178.7 (5)	N1—C7—O1—C8	1.0 (5)
C1—C6—C7—N1	0.1 (6)	C6—C7—O1—C8	−178.7 (4)
C5—C6—C7—O1	−1.6 (8)	C17—C8—O1—C7	1.4 (5)
C1—C6—C7—O1	179.7 (4)	C9—C8—O1—C7	−178.4 (5)
C17—C8—C9—C10	0.6 (8)	C4—C3—O2—C18	−2.7 (8)
O1—C8—C9—C10	−179.6 (5)	C2—C3—O2—C18	178.5 (5)
C8—C9—C10—C11	3.2 (8)	N2—C25—O3—C26	1.3 (5)
C9—C10—C11—C12	176.0 (6)	C24—C25—O3—C26	−173.7 (4)
C9—C10—C11—C16	−1.7 (8)	C35—C26—O3—C25	−0.5 (5)
C10—C11—C12—C13	−173.7 (6)	C27—C26—O3—C25	178.8 (5)
C16—C11—C12—C13	4.1 (9)	C22—C21—O4—C36	−11.5 (7)
C11—C12—C13—C14	0.3 (10)	C20—C21—O4—C36	167.2 (4)
C12—C13—C14—C15	−3.6 (10)	C39—C38—O5—Ir1	2.2 (7)
C13—C14—C15—C16	2.3 (9)	C37—C38—O5—Ir1	−177.6 (3)
C14—C15—C16—C17	177.7 (5)	C1—Ir1—O5—C38	−179.5 (4)
C14—C15—C16—C11	2.1 (7)	N1—Ir1—O5—C38	−98.6 (4)
C12—C11—C16—C15	−5.2 (7)	N2—Ir1—O5—C38	88.6 (4)
C10—C11—C16—C15	172.5 (5)	O6—Ir1—O5—C38	1.0 (3)
C12—C11—C16—C17	178.8 (5)	C39—C40—O6—Ir1	5.7 (6)
C10—C11—C16—C17	−3.5 (7)	C41—C40—O6—Ir1	−173.6 (3)
O1—C8—C17—N1	−3.1 (5)	C19—Ir1—O6—C40	173.3 (3)
C9—C8—C17—N1	176.7 (5)	N1—Ir1—O6—C40	82.7 (3)
O1—C8—C17—C16	174.2 (4)	N2—Ir1—O6—C40	−105.6 (3)
C9—C8—C17—C16	−6.0 (7)	O5—Ir1—O6—C40	−4.7 (3)
C15—C16—C17—C8	−168.7 (5)	O1—C7—N1—C17	−2.9 (5)
C11—C16—C17—C8	7.0 (6)	C6—C7—N1—C17	176.8 (4)
C15—C16—C17—N1	7.8 (8)	O1—C7—N1—Ir1	177.7 (3)
C11—C16—C17—N1	−176.5 (5)	C6—C7—N1—Ir1	−2.6 (5)
C1—Ir1—C19—C20	87.0 (4)	C8—C17—N1—C7	3.6 (5)
N1—Ir1—C19—C20	6.1 (4)	C16—C17—N1—C7	−173.2 (5)
N2—Ir1—C19—C20	178.4 (4)	C8—C17—N1—Ir1	−177.4 (4)
O6—Ir1—C19—C20	−93.3 (4)	C16—C17—N1—Ir1	5.8 (9)
C1—Ir1—C19—C24	−98.6 (3)	C1—Ir1—N1—C7	3.0 (3)
N1—Ir1—C19—C24	−179.5 (3)	C19—Ir1—N1—C7	97.8 (3)
N2—Ir1—C19—C24	−7.1 (3)	N2—Ir1—N1—C7	55.6 (8)
O6—Ir1—C19—C24	81.1 (3)	O6—Ir1—N1—C7	−171.5 (3)
C24—C19—C20—C21	−1.2 (6)	O5—Ir1—N1—C7	−85.4 (3)
Ir1—C19—C20—C21	173.2 (3)	C1—Ir1—N1—C17	−175.9 (5)
C19—C20—C21—O4	−179.5 (4)	C19—Ir1—N1—C17	−81.1 (5)
C19—C20—C21—C22	−0.8 (7)	N2—Ir1—N1—C17	−123.4 (7)
O4—C21—C22—C23	−179.0 (4)	O6—Ir1—N1—C17	9.6 (5)
C20—C21—C22—C23	2.4 (7)	O5—Ir1—N1—C17	95.6 (5)
C21—C22—C23—C24	−1.9 (7)	O3—C25—N2—C35	−1.5 (5)
C22—C23—C24—C25	177.6 (4)	C24—C25—N2—C35	173.5 (4)
C22—C23—C24—C19	−0.1 (7)	O3—C25—N2—Ir1	−176.0 (3)
C20—C19—C24—C23	1.7 (6)	C24—C25—N2—Ir1	−1.1 (5)
Ir1—C19—C24—C23	−173.6 (4)	C26—C35—N2—C25	1.1 (5)
C20—C19—C24—C25	−176.3 (4)	C34—C35—N2—C25	−177.7 (5)
Ir1—C19—C24—C25	8.4 (5)	C26—C35—N2—Ir1	172.0 (4)
C23—C24—C25—N2	177.4 (4)	C34—C35—N2—Ir1	−6.8 (9)
C19—C24—C25—N2	−4.7 (6)	C1—Ir1—N2—C25	99.0 (3)
C23—C24—C25—O3	−8.1 (7)	C19—Ir1—N2—C25	4.5 (3)
C19—C24—C25—O3	169.8 (4)	N1—Ir1—N2—C25	47.4 (8)
C35—C26—C27—C28	0.6 (8)	O6—Ir1—N2—C25	−86.4 (3)
O3—C26—C27—C28	−178.6 (5)	O5—Ir1—N2—C25	−172.5 (3)
C26—C27—C28—C29	−0.8 (8)	C1—Ir1—N2—C35	−71.7 (5)
C27—C28—C29—C30	−178.8 (5)	C19—Ir1—N2—C35	−166.3 (5)
C27—C28—C29—C34	1.2 (8)	N1—Ir1—N2—C35	−123.4 (7)
C28—C29—C30—C31	−179.8 (5)	O6—Ir1—N2—C35	102.9 (5)
C34—C29—C30—C31	0.2 (8)	O5—Ir1—N2—C35	16.8 (5)

### Hydrogen-bond geometry (Å, °)

**Table:**

*D*—H···*A*	*D*—H	H···*A*	*D*···*A*	*D*—H···*A*
C15—H15A···O6	0.93	2.24	3.077 (5)	150
C33—H33A···O5	0.93	2.22	3.137 (6)	170
C23—H23A···O6^i^	0.93	2.54	3.419 (6)	157
C27—H27A···O2^ii^	0.93	2.55	3.206 (6)	128

Symmetry codes: (i) −*x*+1, −*y*+2, −*z*; (ii) −*x*+1, −*y*+1, −*z*.
